# Gambling behaviour of Bradford teenagers: evidence from the Born in Bradford Age of Wonder study

**DOI:** 10.12688/wellcomeopenres.25913.2

**Published:** 2026-08-17

**Authors:** Gillian Santorelli, Jane West, John Pickavance

**Affiliations:** 1 Born in Bradford, Bradford Institute for Health Research, Bradford, England, BD9 6RJ, UK; 2 Bradford Centre for Health Data Science, Bradford Institute for Health Research, Bradford, England, BD9 6RJ, UK

**Keywords:** Adolescent gambling, Ethnicity, Born in Bradford, Age of Wonder, Public health

## Abstract

**Background:**

Gambling among young people is a public health concern, and gambling-like mechanics in digital games (e.g., loot boxes) may increase exposure. We estimated the prevalence of traditional gambling and gambling-like online behaviours among adolescents in Bradford.

**Methods:**

Born in Bradford’s Age of Wonder survey (2023–24) collected self-reported gambling behaviours from pupils in Years 8–10 (ages 12–15) across secondary schools. Invalid responses were excluded, yielding analytic samples for traditional gambling (n=7,257) and online gaming behaviours (n=7,828). Prevalence was summarised by year group, sex, and ethnicity (Pakistani British, White British, Other).

**Results:**

Of 7,254 pupils, 28.7% reported ever spending money on at least one gambling activity. Prevalence was similar for males (29.6%) and females (28.0%) and across year groups (Year 8: 30.8%; Year 9: 28.1%; Year 10: 27.1%). The most common activity was fruit/slot machines (16.7%), followed by private bets (7.9%) and playing cards for money (6.7%). Marked ethnic differences were observed: White British pupils were more likely to report ever gambling (45.8%) than Pakistani British pupils (15.8%) and pupils of other ethnicities (24.1%).

Among 7,825 pupils answering online gaming questions, 55.8% had spent real money in at least one online game, 27.2% had purchased loot boxes/packs, and 3.3% had bet with in-game items. Males reported higher in-game spending than females (73.2% vs 39.8%), and White British pupils reported higher spending than Pakistani British pupils (66.8% vs 46.3%).

**Conclusions:**

Gambling and gambling-like behaviours are common among Bradford adolescents, with substantial demographic variation, particularly by ethnicity and sex. Findings support culturally sensitive, targeted public health and school-based prevention strategies addressing both traditional gambling and gambling-like online mechanisms.

## Introduction

Gambling among children and young people is a significant public health challenge despite legal age restrictions. The 2024 Gambling Commission Young People and Gambling Survey found that 27% of 11–17-year-olds had spent their own money on any gambling activity in the last 12 months. ^1^ Playing arcade gaming machines and placing a bet for money with family and friends were reported to be the most common forms. ^1^ Research has shown that exposure to gambling advertising, family gambling behaviours, and the gamification of digital entertainment all contribute to the normalisation of gambling. ^2– 4^ Young people who gamble face higher risks of developing problem gambling behaviours and associated harms including financial difficulties, mental health issues, and educational disruption. ^1, 5, 6^ Whilst regulatory bodies have strengthened some protections, concerns remain about the blurring of boundaries between gaming and gambling through loot boxes and social casino games that may serve as a first step towards traditional gambling for young people. Preventing harm requires reliable and timely prevalence data to identify those young people at greatest risk of harm and to help support policy decisions and prevention strategies. Born in Bradford’s Age of Wonder study ^7^ provides the opportunity to gain some insight into gambling behaviour among young people living in diverse and mostly disadvantaged communities in Bradford. This data note forms part of an overview of the health and lifestyle of adolescents in Bradford, using quantitative data from the Age of Wonder study. ^7^

## Methods

We conducted a cross-sectional analysis of Born in Bradford’s Age of Wonder study ^7^ survey completed in the academic year 2023/24. We invited 36 secondary schools across the Bradford district to participate, of which 25 took part; pupils from Years 8–10 (age 12–15 years) in each school completed the questionnaire. The gambling questions in the survey capture information on gambling activity, spend, and online games.

Participating schools provided demographic data including date of birth, postcode, year group, sex, ethnicity, free school meals status, and special educational needs status. Where ethnicity was missing, we used data from the self-report Age of Wonder survey. Ethnicity was categorised using Office for National Statistics census group classification 6b. ^8^

### Data cleaning and sample definition

Prior to analysis, participants with invalid responses were excluded. This included those who had failed to answer any of the “spend money on gambling” checkboxes or who selected all options in the affirmative. Selecting all options was considered implausible and indicative of non-differentiated response (i.e, selecting all items without regard to content), a recognised indicator of low-quality survey data, which can bias prevalence estimates upwards in self-reported surveys. ^9^ The same criteria were applied to the questions regarding online games. Following these procedures, two analytic samples were defined: traditional gambling and online gaming.

### Measures

The gambling module was designed to capture multiple aspects of gambling behaviour. Participants were asked if they had ever spent their own money on any of ten specific gambling activities, followed by a question on the recency of any endorsed activities. The module also captured data on engagement with gambling-like features in online games, such as purchasing ‘loot boxes’ or similar randomised reward mechanisms, or betting using in-game items. The survey questions and their corresponding response options are detailed in Table 1.

**Table 1.** Gambling and Online Gaming Survey Measures.

Topic	Survey question	Response options
**Traditional gambling activities**	Have you ever spent money on any of the following activities? Please tick all that apply.	1. Lotto (the main National Lottery draw), National Lottery Scratchcards which you bought in a shop (not free Scratchcards). Any other National Lottery games (e.g., Euromillions, Thunderball, HotPicks, Set For Life). Other Lotteries (e.g., The Health Lottery, People’s Postcode Lottery, or any other smaller lotteries) 2. Fruit or slot machines (e.g., at an arcade, pub, or social club) 3. Placing a private bet for money (e.g., with friends or family) 4. Playing cards for money (e.g., with friends or family) 5. Bingo at a club 6. Bingo at somewhere other than a bingo club (e.g., social club, holiday park, etc.) 7. Personally visiting a betting shop to play gaming machines 8. Personally placing a bet at a betting shop (e.g., on football, dog racing or horse racing) 9. Personally visiting a casino to play casino games (by this we mean a proper casino with roulette tables) can win real money (e.g., poker, casinos, bingo, betting on sport or racing) 10. Gambling websites/apps where you can win real money (e.g., poker, casinos, bingo, betting on sport or racing) 11. None of these
**Recency of gambling**	*(Asked for each endorsed activity above)*: When did you last spend money on [Activity]?	1. In the last 7 days 2. In the last month 3. In the last year 4. More than a year ago
**Online gaming & loot boxes**	In which, if any, of the following ways have you personally ever used in-game items or currency? Tick all that apply	1. Paid money (or used virtual currency you have bought) to buy specific in-game items (e.g., skins, clothes, weapons, players) 2. Paid money (or used virtual currency you have bought) to open loot boxes/packs/chests to get other in-game items (e.g., skins, clothes, weapons, players) 3. Bet with in-game items on websites outside of the game you are playing 4. None of these

### Ethical approval and consent

The Age of Wonder study is approved by the Bradford Leeds NHS Research Ethics Committee [Ref: 21/YH/0261, date: 22.12.21]. Parents received information sheets about surveys, and provided written opt-out consent in advance if they did not want their child to participate. Young people provided their verbal assent on the day. Verbal assent was obtained on the basis that young people aged 12-15 are unable to legally provide their own consent but must agree to participate.

### Analysis

We calculated the prevalence of each gambling behaviour overall and stratified by age group, sex, and academic year group. Given the demographic profile of Bradford, ethnic group analyses were stratified into the city’s primary groups: Pakistani British, White British, and ‘Other’ (comprising all other ethnic groups). This approach reflects the fact that the majority of pupils classified as Asian/Asian British were of Pakistani heritage. Prevalence estimates are presented with 95% confidence intervals calculated using binomial exact methods. Given the large sample size, interpretation emphasised effect sizes and confidence intervals rather than statistical significance.

## Results

In total, 9,003 pupils completed the Age of Wonder questionnaire in the 2023-24 academic year, with a median of 342 participants per school (IQR 287-381). This represents an overall response rate of 40.9% when calculated against all pupils at the 36 invited schools.

As shown in Table 2, the demographic profile of the sample reflects Bradford’s diverse and deprived population. Pupils of Asian/Asian British ethnicity constituted 47.5% of the sample (comprising 40.0% of Pakistani heritage and 7.5% other Asian/Asian British); 40% were of White ethnicity (35.4% White British, 4.6% White Other), and 3.7% were Black/Black British.

**Table 2.** Demographic characteristics of the 9003 young people who completed the Age of Wonder survey in the 2023/24 academic year.

Variable	Level	N (%)	Percentage of all secondary school pupils in Bradford local authority (Y8-10), 2023/24	Percentage of all secondary school pupils in England, 2023/24 (for comparison)
Total		9003 (100)	-	-
Age in years [IQR]		13.9 [13.2-14.7]	-	-
Academic year group	Year 8	3168 (35.2)	33.4	-
	Year 9	3212 (35.7)	33.5	-
	Year 10	2623 (29.1)	33.0	-
Sex ^a^	Male	4292 (47.7)	50.5	50.7
	Female	4711 (52.3)	49.5	49.3
Ethnicity ^b^	Asian and Asian British	4276 (47.5)	44.2	13.4
	* Pakistani*	*3598 (40.0)*	37.3	*4.6*
	*Other Asian*	*678 (7.5)*	6.9	*8.8*
	White	3599 (40.0)	43.1	68.9
	*White British*	*3186 (35.4)*	37.7	*61.4*
	*Other White*	*413 (4.6)*	5.5	*7.5*
	Black and Black British	333 (3.7)	2.9	6.7
	Mixed ethnicities	469 (5.2)	5.8	6.7
	Other	153 (1.7)	1.8	2.4
	Missing/unknown	173 (1.9)	2.1	2.0
Free school meals ^c^		2569 (28.5)	31.9	25.7
IMD quintile of home address ^d^	1 (most deprived)	5548 (61.6)	53.6	23.7
	2	1796 (19.9)	16.5	20.0
	3	637 (7.1)	16.1	18.5
	4	425 (4.7)	7.9	18.2
	5 (least deprived)	590 (6.6)	5.9	19.7
	Missing	7 (0.1)	-	-
SEN provision ^e^		1273 (14.1)	18.1	16.3

Data for the comparison columns are from the Department for Education 2023/24 school census for state-funded secondary schools, accessed via https://explore-education-statistics.service.gov.uk/.

^a^ school years 8-10;

^b^ school years 7-11,

^c^ school years 8-10;

^d^ calculated from population of 12-15 year-olds, based on mid-2022 population estimates for of Lower Level Super Output Areas in England ( https://www.ons.gov.uk/peoplepopulationandcommunity/populationandmigration/populationestimates/datasets/lowersuperoutputareamidyearpopulationestimates) and IMD 2019 quintiles of LSOAs ( https://www.gov.uk/government/statistics/english-indices-of-deprivation-2019);

^e^ EHC plans or SEN support for pupils in school years 8-10.

The final analytic sample for the “ever spent money on gambling” questions consists of 7,257 pupils; for those who answered questions about online gaming, it was 7,828. Table 3 provides the demographic summaries of these samples, which were similar to the overall Age of Wonder questionnaire sample.

**Table 3.** Demographic characteristics of the young people who completed the gambling questions on (1) ever spent money on gambling, and (2) plays online games in Age of Wonder survey in the 2023/24 academic year.

Variable	Level	N (%) who answered questions on ‘ever spent money on gambling’	N (%) who answered questions on online games
Total		7254 (100)	7825 (100)
Age in years [IQR]		14.0 [13.0-14.0]	14.0 [13.0-14.0]
Academic year group	Year 8	2496 (34.4)	2684 (34.3)
	Year 9	2581 (35.6)	2805 (35.9)
	Year 10	2177 (30.0)	2336 (29.9)
Sex	Male	3368 (46.4)	3735 (47.7)
	Female	3886 (53.6)	4090 (52.3)
Ethnicity	Asian and Asian British	3382 (46.6)	3719 (47.5)
	* Pakistani*	*2823 (38.9)*	*3102 (39.6)*
	*Other Asian*	*559 (7.7)*	*616 (7.9)*
	White	3599 (40.0)	3134 (40.1)
	*White British*	*2626 (36.2)*	*2773 (35.4)*
	*Other White*	*335 (4.6)*	*351 (4.5)*
	Black and Black British	270 (3.7)	289 (3.7)
	Mixed ethnicities	377 (5.2)	412 (5.3)
	Other	115 (1.6)	124 (1.6)
	Missing/unknown	141 (1.9)	148 (1.9)
	*Other*	*911 (12.6)*	*983 (12.6)*

### Overall gambling prevalence

Among 7,257 pupils who responded to the Age of Wonder survey gambling questions, 28.7% reported having ever spent money on at least one gambling activity. The most common activity was playing on fruit or slot machines (16.7%), placing private bets for money (7.9%), and playing cards for money (6.7%).

### Demographic differences in gambling behaviour

Figures 1– 11 present the prevalence of gambling behaviour stratified by demographic profiles. Table 4 shows the prevalence and recency of participation in traditional gambling activities, with denominators and confidence intervals provided in Table 5. The prevalence of any form of gambling was higher in Year 8 pupils (30.8%) compared to those in Year 9 (28.1%), and Year 10 (27.1%). Different types of gambling behaviour were also broadly similar across years groups, though Year 8 pupils were more likely to play on slot machines.

**Figure 1.**
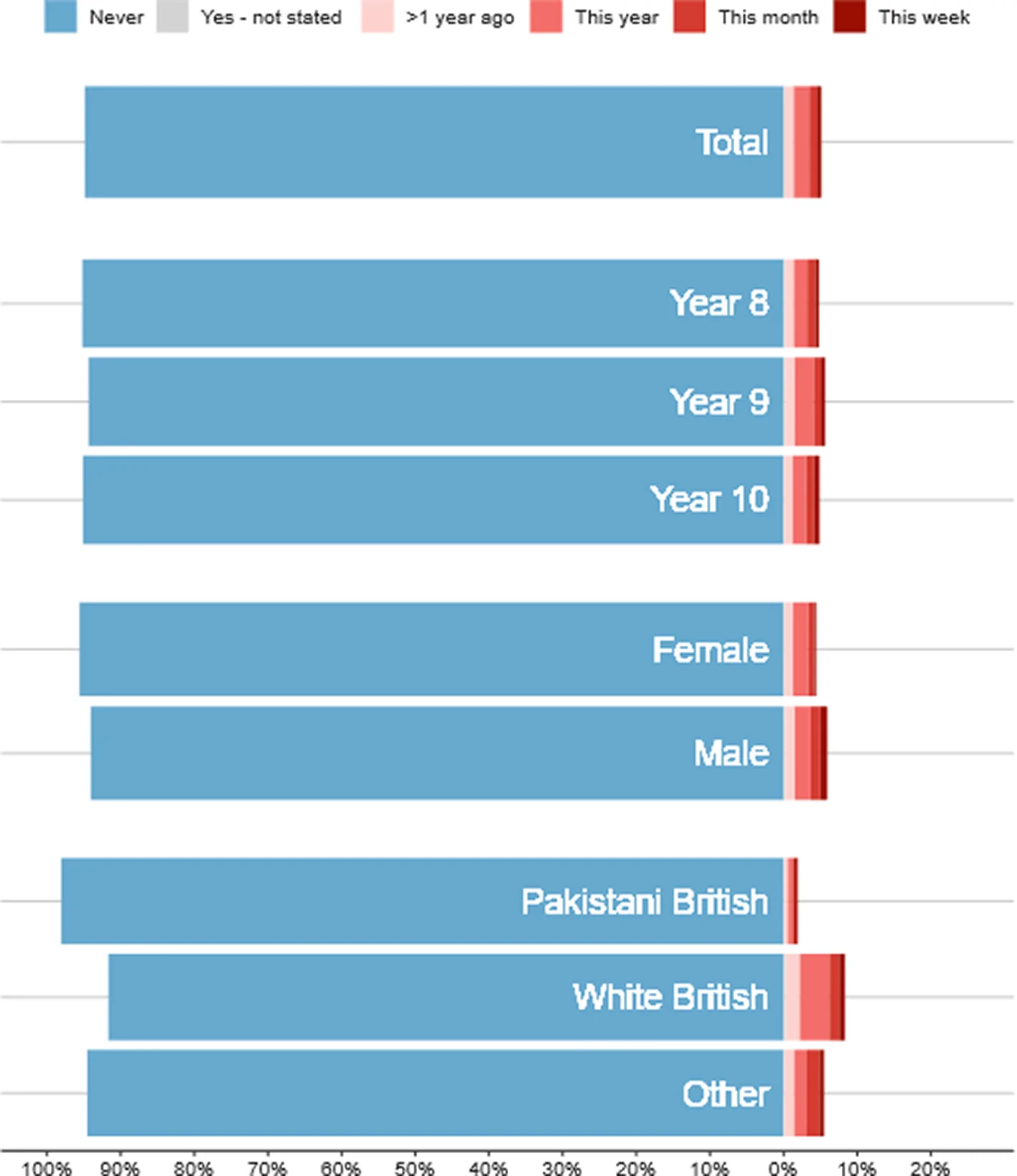
Last spent money on Lotto, National Lottery Scratchcards, or other National Lottery games.

**Figure 2.**
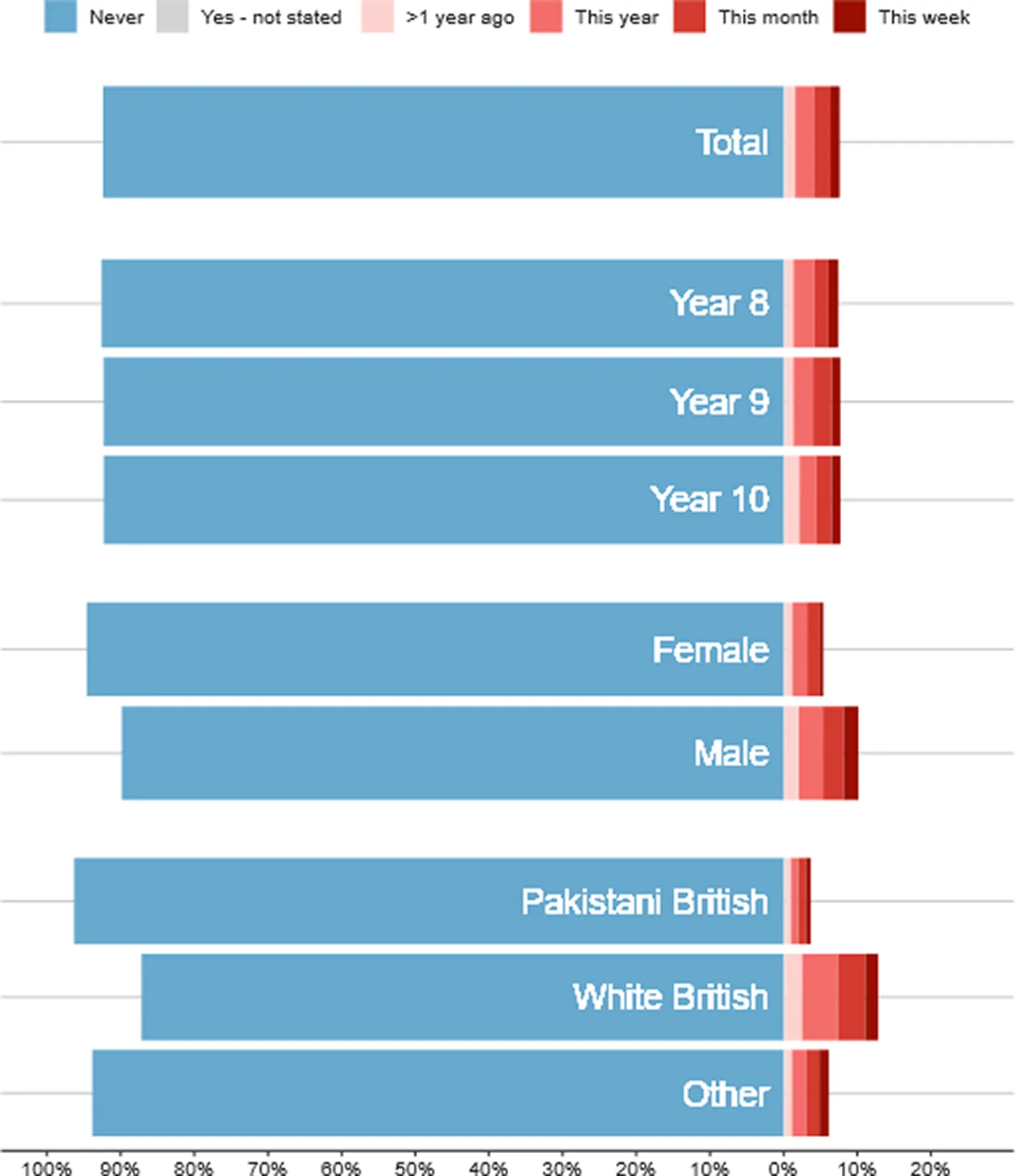
Last placed a private bet for money.

**Figure 3.**
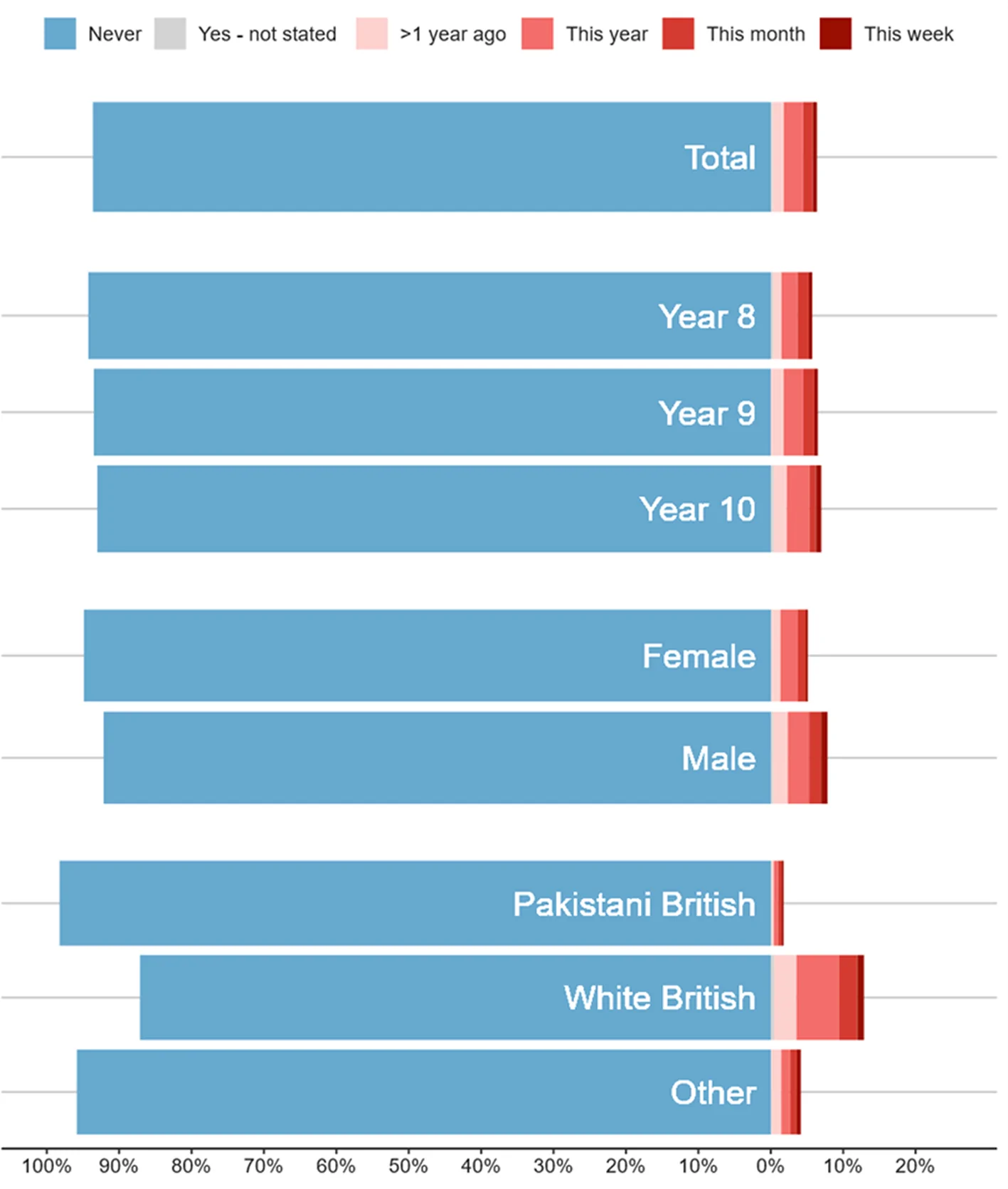
Last spent money on playing cards for money.

**Figure 4.**
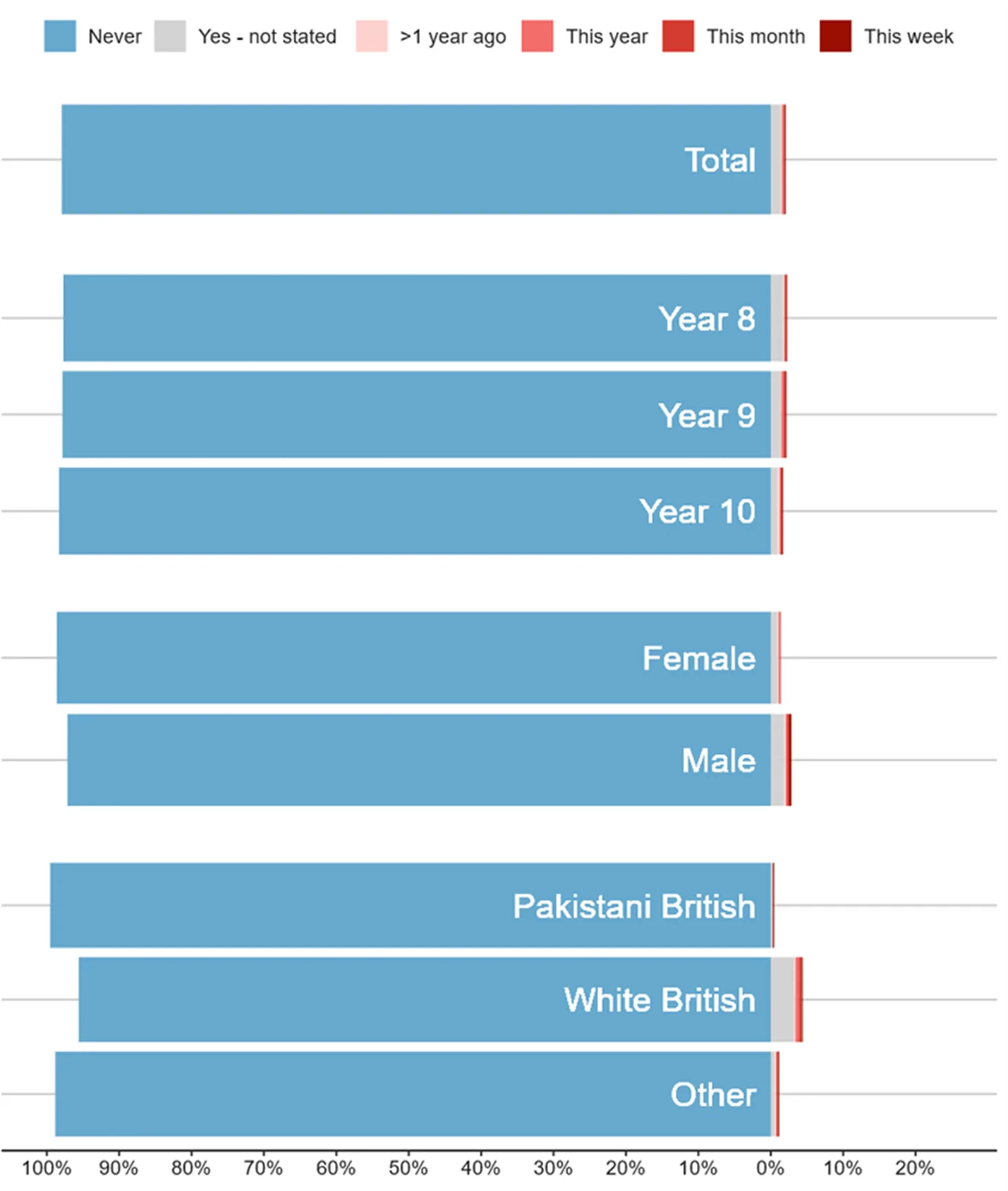
Last spent money placing a bet in a betting shop.

**Figure 5.**
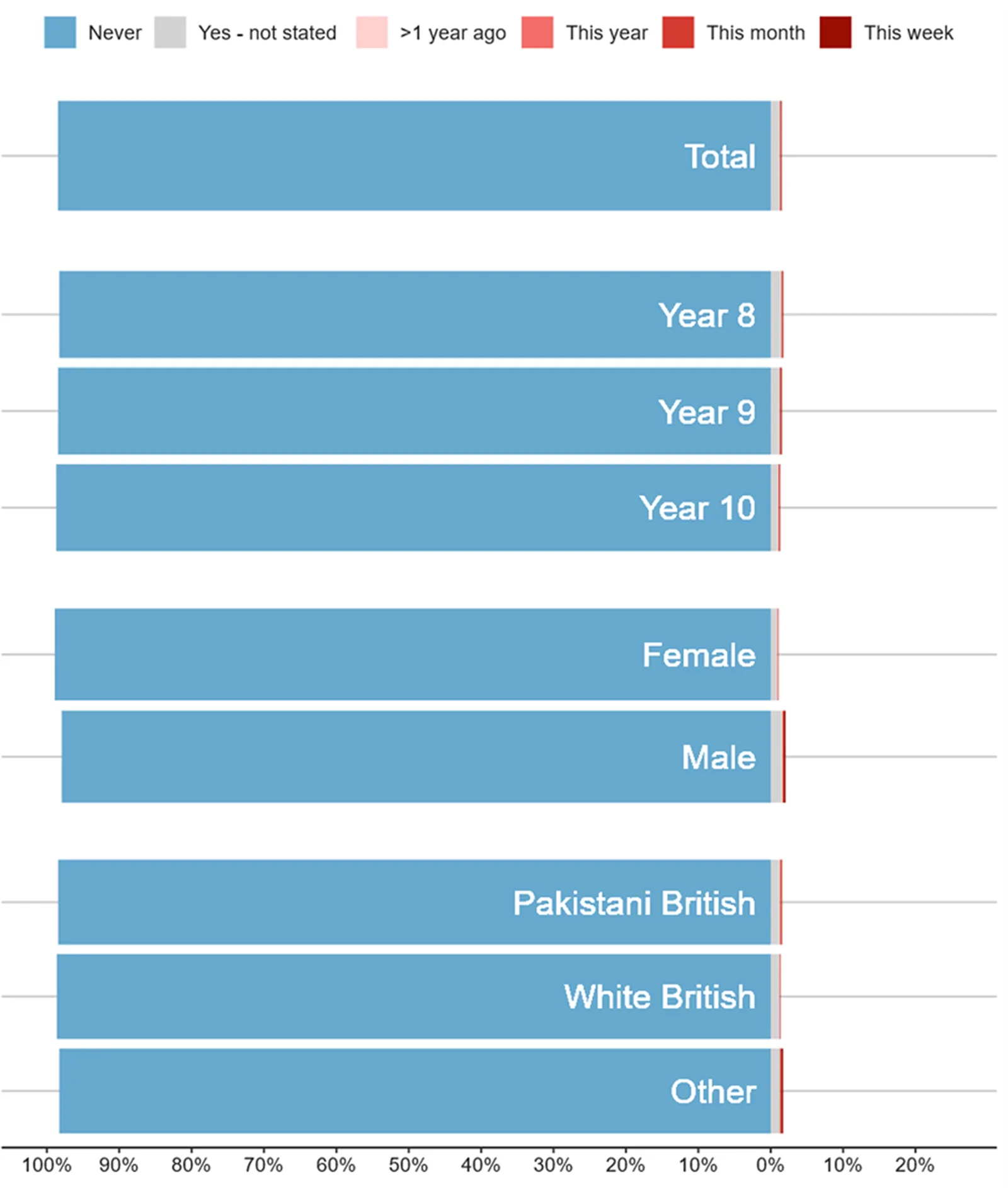
Last spent money visiting a betting shop to play gaming machines.

**Figure 6.**
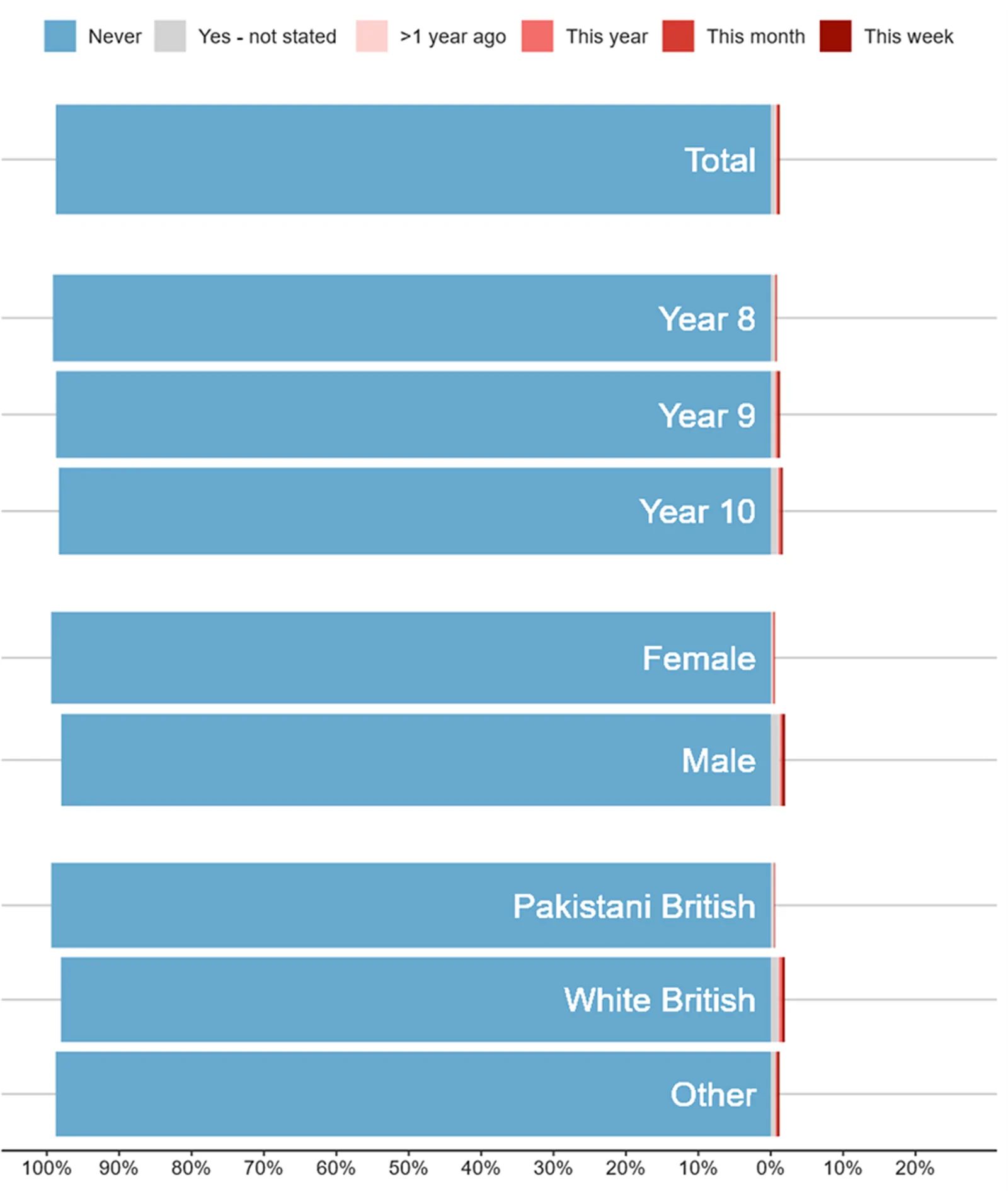
Last spent money on gambling websites/apps where you can win real money.

**Figure 7.**
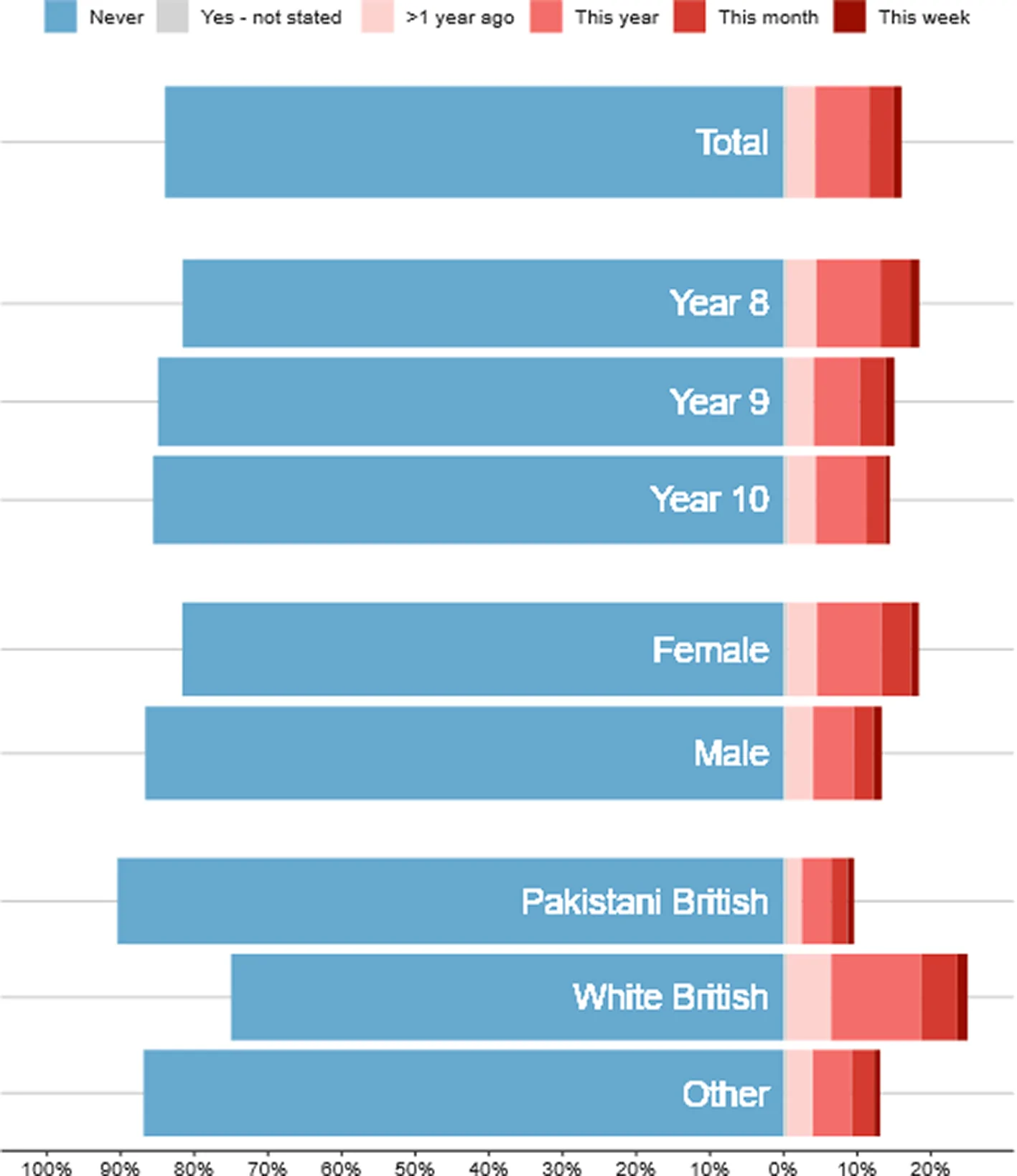
Last spent money on fruit or slot machines.

**Figure 8.**
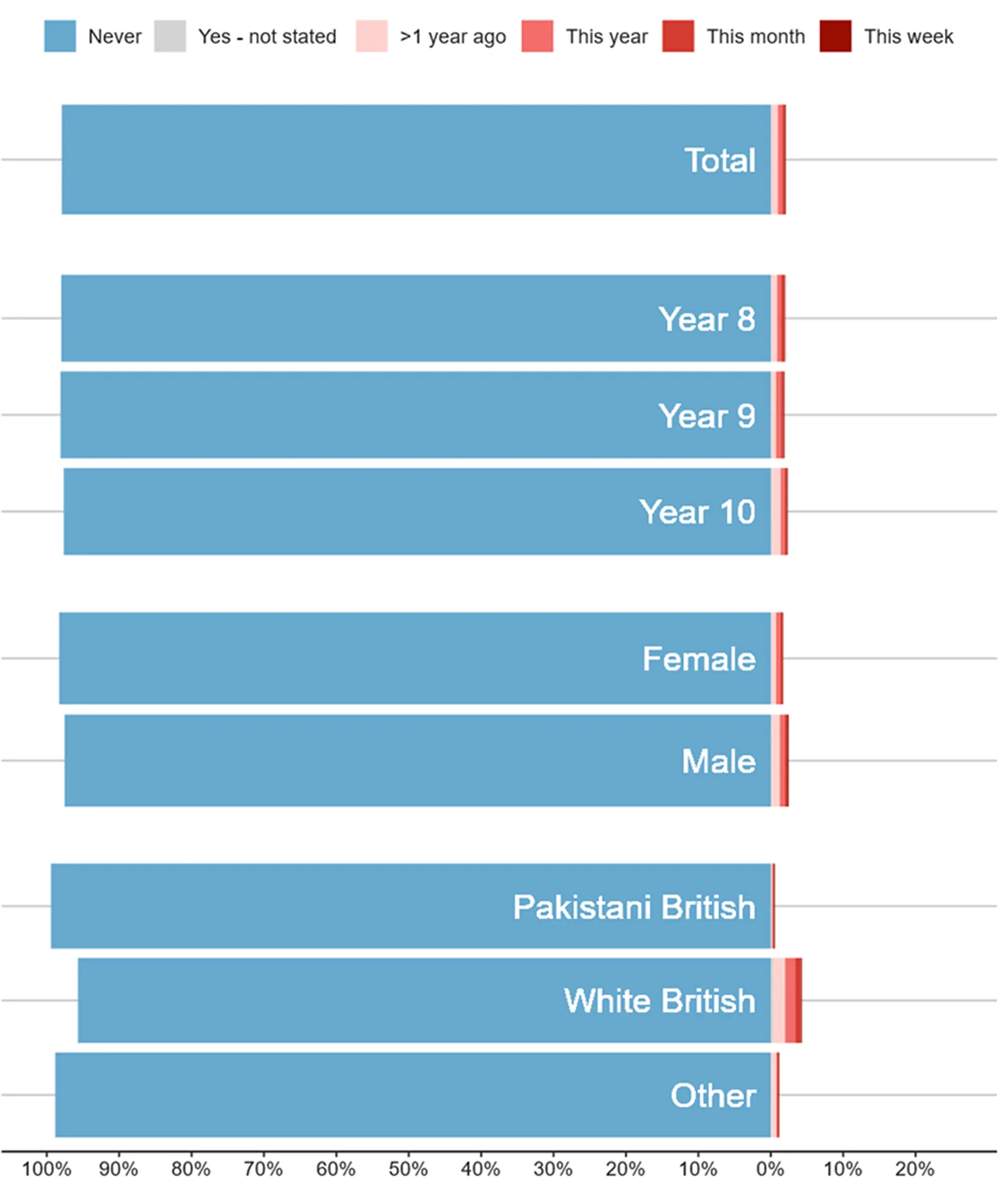
Last spent money on bingo at a bingo club.

**Figure 9.**
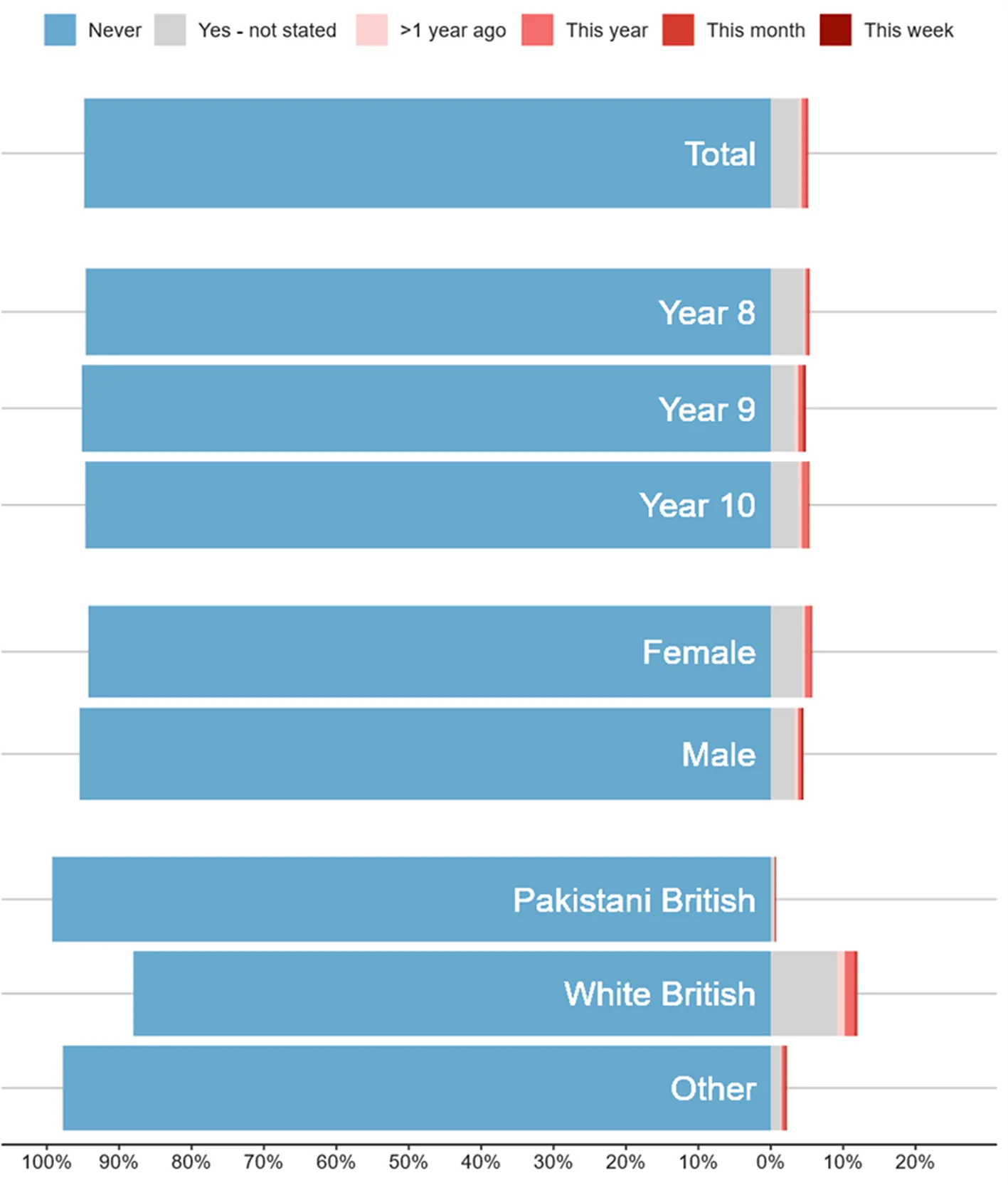
Last spent money on bingo at somewhere other than a bingo club.

**Figure 10.**
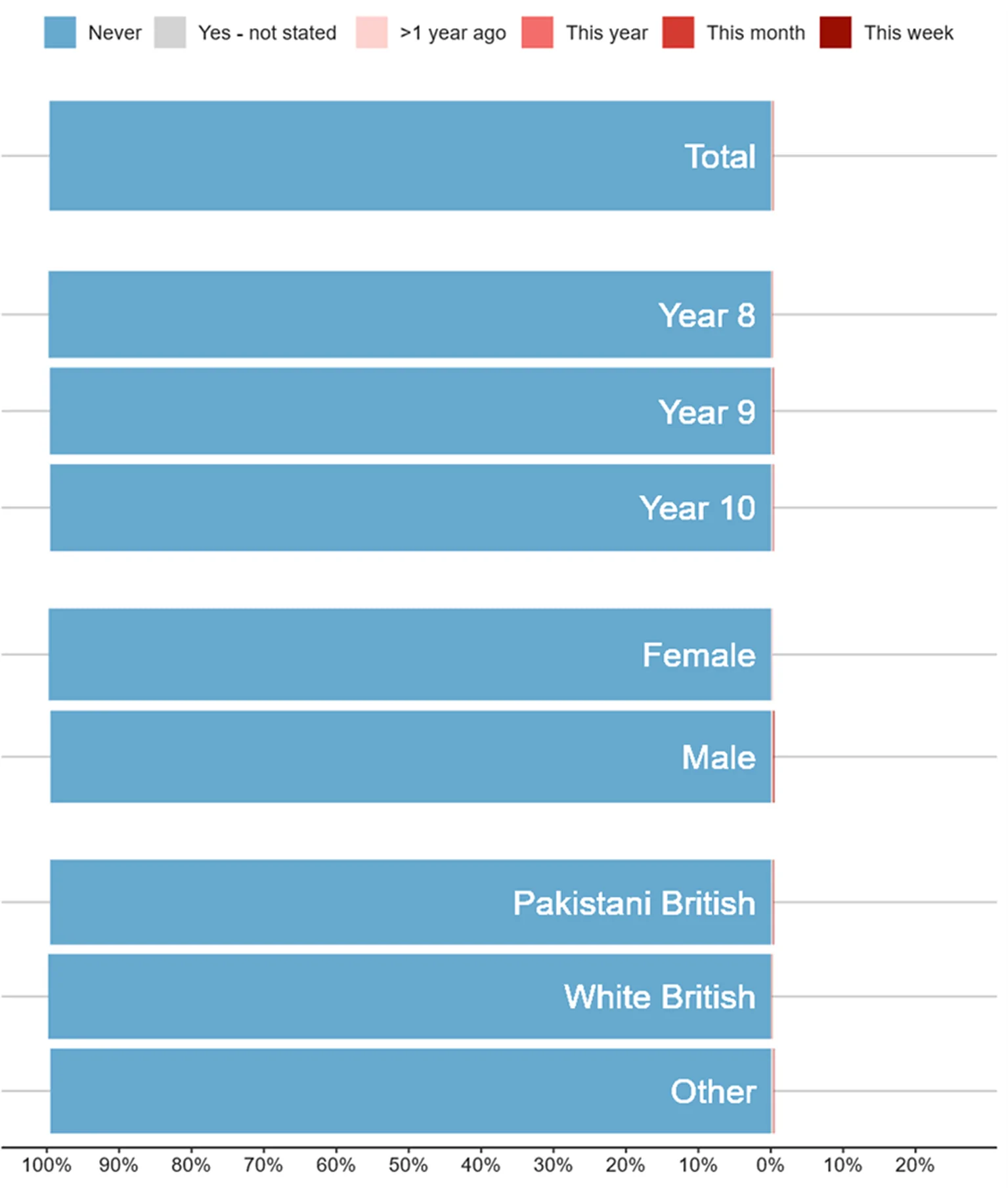
Last spent money visiting a casino to play casino games.

**Figure 11.**
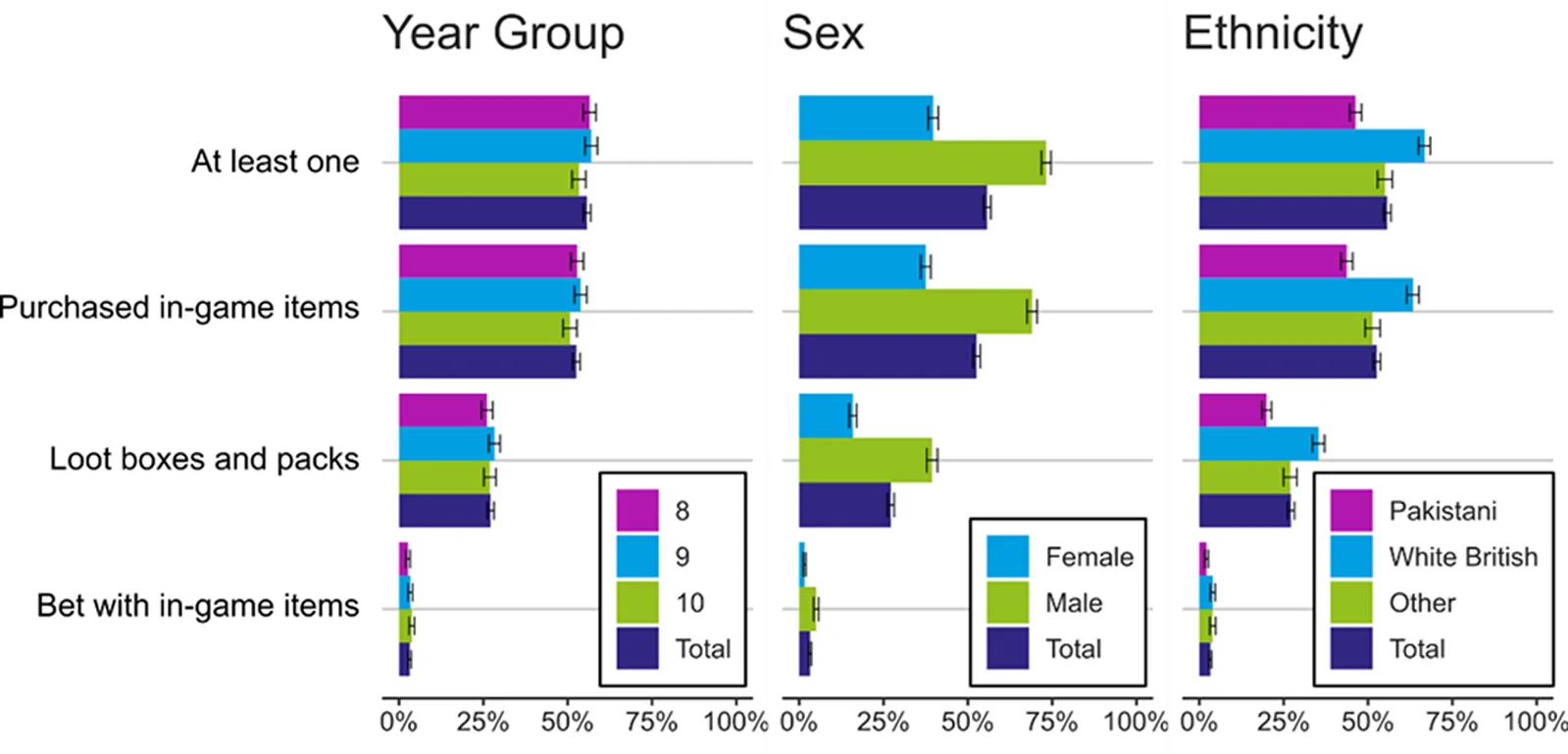
Prevalence of spending money within online games.

**Table 4.** Recency of participation in traditional gambling activities among pupils who reported ever gambling, by school year group, sex, and ethnicity.

		Fruit or slot machines						Private bets						Playing cards						Bingo (elsewhere)						National Lottery games					
		Never	Yes – NS	>1 year ago	This year	This month	This week	Never	Yes – NS	>1 year ago	This year	This month	This week	Never	Yes – NS	>1 year ago	This year	This month	This week	Never	Yes – NS	>1 year ago	This year	This month	This week	Never	Yes – NS	>1 year ago	This year	This month	This week
** Total**	**Total**	**84.0**	**0.5**	**3.8**	**7.3**	**3.4**	**1.0**	**92.4**	**0.3**	**1.3**	**2.6**	**2.2**	**1.2**	**93.6**	**0.2**	**1.6**	**2.7**	**1.4**	**0.6**	**94.8**	**3.9**	**0.4**	**0.6**	**0.2**	n < 10	**94.9**	**0.2**	**1.2**	**2.1**	**1.1**	**0.6**
Year Group	8	81.6	0.4	4.0	8.7	4.0	1.2	92.6	n < 10	1.0	2.8	1.9	1.3	94.3	n < 10	1.2	2.2	1.6	0.5	94.6	4.6	n < 10	n < 10	n < 10	n < 10	95.2	n < 10	1.2	1.8	1.1	0.5
	9	84.9	0.4	3.7	6.2	3.6	1.2	92.3	n < 10	1.1	2.6	2.6	1.2	93.5	n < 10	1.7	2.7	1.5	0.5	95.2	3.3	0.5	0.5	n < 10	n < 10	94.3	n < 10	1.3	2.6	1.0	0.6
	10	85.6	0.6	3.8	6.9	2.5	0.6	92.3	n < 10	1.9	2.3	2.1	1.2	93.0	n < 10	1.8	3.1	0.9	0.7	94.7	3.8	0.5	0.8	n < 10	n < 10	95.1	n < 10	1.1	1.8	1.1	0.7
Sex	Female	81.6	0.6	4.0	8.7	4.1	1.0	94.6	n>=10	0.8	2.0	1.7	0.5	94.9	n < 10	1.2	2.4	1.1	0.3	94.3	4.4	0.3	0.7	n < 10	n < 10	95.6	n < 10	1.2	2.1	0.8	0.3
	Male	86.7	0.4	3.6	5.6	2.7	1.1	89.8	n < 10	1.9	3.3	2.8	2.0	92.2	n>=10	2.0	2.9	1.7	0.9	95.5	3.3	0.5	0.3	n < 10	n < 10	94.1	n < 10	1.2	2.1	1.3	1.0
Ethnicity	Pakistani British	90.4	n < 10	2.0	4.0	2.2	0.9	96.3	n < 10	0.7	1.0	1.0	0.7	98.2	n < 10	0.3	0.6	0.5	0.2	99.3	0.5	n < 10	n < 10	n < 10	n < 10	98.1	n < 10	0.5	0.6	0.4	0.4
	White British	75.0	n < 10	6.0	12.2	4.9	1.4	87.2	n < 10	2.2	4.8	3.7	1.8	87.1	n>=10	3.1	5.9	2.5	0.9	88.0	9.2	1.0	1.3	n < 10	n < 10	91.7	n < 10	1.9	4.0	1.3	0.8
	Other	86.9	n < 10	3.5	5.3	3.1	0.8	93.9	n < 10	0.9	1.9	1.8	1.3	95.8	n < 10	1.3	1.2	0.9	0.6	97.8	1.4	n < 10	n < 10	n < 10	n < 10	94.5	n < 10	1.3	1.7	1.7	0.6

		Placing a bet						Bingo (club)						Gaming machines						Gambling websites/apps						Casino					
		Never	Yes – NS	>1 year ago	This year	This month	This week	Never	Yes – NS	>1 year ago	This year	This month	This week	Never	Yes – NS	>1 year ago	This year	This month	This week	Never	Yes – NS	>1 year ago	This year	This month	This week	Never	Yes – NS	>1 year ago	This year	This month	This week
**Total**	** Total**	**97.9**	**1.4**	**0.2**	**0.2**	**0.2**	**0.2**	**97.9**	**0.1**	**0.8**	**0.6**	**0.4**	**0.1**	**98.5**	**1.2**	n < 10	n < 10	n < 10	n < 10	**98.8**	n < 10	n < 10	**0.2**	n < 10	**0.2**	**99.6**	**0.2**	n < 10	n < 10	n < 10	n < 10
Year Group	8	97.7	1.8	n < 10	n < 10	n < 10	n < 10	98.0	n < 10	0.8	0.5	0.5	n < 10	98.3	1.4	n < 10	n < 10	n < 10	n < 10	99.2	n < 10	n < 10	0.0	n < 10	0.1	99.8	n < 10	n < 10	n < 10	n < 10	n < 10
	9	97.8	1.4	n < 10	n < 10	n < 10	n < 10	98.1	n < 10	0.6	0.7	n < 10	n < 10	98.5	1.2	n < 10	n < 10	n < 10	n < 10	98.7	n < 10	n < 10	0.2	n < 10	0.3	99.6	n < 10	n < 10	n < 10	n < 10	n < 10
	10	98.3	1.0	n < 10	n < 10	n < 10	n < 10	97.7	n < 10	1.2	0.6	n < 10	n < 10	98.7	0.9	n < 10	n < 10	n < 10	n < 10	98.3	n < 10	n < 10	0.2	n < 10	0.2	99.5	n < 10	n < 10	n < 10	n < 10	n < 10
Sex	Female	98.6	1.0	n < 10	n < 10	n < 10	n < 10	98.3	n < 10	0.6	0.5	0.4	n < 10	98.9	0.9	n < 10	n < 10	n < 10	n < 10	99.4	n < 10	n < 10	0.2	n < 10	0.1	99.7	n < 10	n < 10	n < 10	n < 10	n < 10
	Male	97.1	1.9	n < 10	n < 10	n < 10	n < 10	97.5	n < 10	1.1	0.6	0.4	n < 10	98.0	1.5	n < 10	n < 10	n < 10	n < 10	98.0	n < 10	n < 10	0.1	n < 10	n>=10	99.5	n < 10	n < 10	n < 10	n < 10	n < 10
Ethnicity	Pakistani British	99.5	n < 10	n < 10	n < 10	n < 10	n < 10	99.4	n < 10	n < 10	n < 10	n < 10	n < 10	98.4	1.2	n < 10	n < 10	n < 10	n < 10	99.4	n < 10	n < 10	0.0	n < 10	0.1	99.5	n < 10	n < 10	n < 10	n < 10	n < 10
	White British	95.6	3.2	n < 10	n>=10	n < 10	n < 10	95.7	n < 10	1.7	1.4	0.8	n < 10	98.6	1.1	n < 10	n < 10	n < 10	n < 10	98.1	n < 10	n < 10	0.3	n < 10	0.3	99.8	n < 10	n < 10	n < 10	n < 10	n < 10
	Other	98.8	n < 10	n < 10	n < 10	n < 10	n < 10	98.8	n < 10	n < 10	n < 10	n < 10	n < 10	98.3	1.2	n < 10	n < 10	n < 10	n < 10	98.8	n < 10	n < 10	0.1	n < 10	0.3	99.5	n < 10	n < 10	n < 10	n < 10	n < 10

Note: Percentages may not sum to 100 due to rounding. Cells with fewer than 10 observations are suppressed in line with disclosure control requirements.

**Table 5.** Detailed prevalence, recency, denominators, and 95% CIs for traditional gambling activities among participants in the Age of Wonder survey, stratified by school year group, sex, and ethnicity.

		Fruit or slot machines						Private bets					
		Never	Yes – NS	>1 year ago	This year	This month	This week	Never	Yes – NS	>1 year ago	This year	This month	This week
** Total**		6094/7257	35/7257	277/7257	527/7257	248/7257	76/7257	6705/7257	20/7257	96/7257	188/7257	159/7257	89/7257
		84.0 (83.1-84.8)	0.5 (0.3-0.6)	3.8 (3.4-4.3)	7.3 (6.7-7.9)	3.4 (3.0-3.8)	1.0 (0.8-1.3)	92.4 (91.8-93.0)	0.3 (0.2-0.4)	1.3 (1.1-1.6)	2.6 (2.2-3.0)	2.2 (1.9-2.5)	1.2 (1.0-1.5)
Year Group	8	2036/2496	11/2496	100/2496	217/2496	101/2496	31/2496	2311/2496	<10	26/2496	70/2496	48/2496	33/2496
		81.6 (80.1-83.1)	0.4 (0.2-0.7)	4.0 (3.2-4.8)	8.7 (7.6-9.8)	4.0 (3.3-4.8)	1.2 (0.8-1.7)	92.6 (91.6-93.6)		1.0 (0.6-1.4)	2.8 (2.2-3.5)	1.9 (1.4-2.5)	1.3 (0.9-1.8)
	9	2194/2583	11/2583	95/2583	160/2583	92/2583	31/2583	2384/2583	<10	28/2583	68/2583	66/2583	30/2583
		84.9 (83.6-86.3)	0.4 (0.2-0.7)	3.7 (2.9-4.4)	6.2 (5.3-7.1)	3.6 (2.9-4.3)	1.2 (0.8-1.6)	92.3 (91.3-93.3)		1.1 (0.7-1.5)	2.6 (2.0-3.3)	2.6 (1.9-3.2)	1.2 (0.8-1.6)
	10	1864/2178	13/2178	82/2178	150/2178	55/2178	14/2178	2010/2178	<10	42/2178	50/2178	45/2178	26/2178
		85.6 (84.1-87.1)	0.6 (0.3-0.9)	3.8 (3.0-4.6)	6.9 (5.8-7.9)	2.5 (1.9-3.2)	0.6 (0.3-1.0)	92.3 (91.2-93.4)		1.9 (1.3-2.5)	2.3 (1.7-2.9)	2.1 (1.5-2.7)	1.2 (0.7-1.7)
Sex	Female	3175/3889	22/3889	155/3889	340/3889	158/3889	39/3889	3679/3889	>=10	33/3889	78/3889	65/3889	21/3889
		81.6 (80.4-82.9)	0.6 (0.3-0.8)	4.0 (3.4-4.6)	8.7 (7.9-9.6)	4.1 (3.4-4.7)	1.0 (0.7-1.3)	94.6 (93.9-95.3)		0.8 (0.6-1.1)	2.0 (1.6-2.4)	1.7 (1.3-2.1)	0.5 (0.3-0.8)
	Male	2919/3368	13/3368	122/3368	187/3368	90/3368	37/3368	3026/3368	<10	63/3368	110/3368	94/3368	68/3368
		86.7 (85.5-87.8)	0.4 (0.2-0.6)	3.6 (3.0-4.3)	5.6 (4.8-6.3)	2.7 (2.1-3.2)	1.1 (0.7-1.4)	89.8 (88.8-90.9)		1.9 (1.4-2.3)	3.3 (2.7-3.9)	2.8 (2.2-3.4)	2.0 (1.5-2.5)
Ethnicity	Pakistani British	2554/2824	>=10	57/2824	112/2824	63/2824	25/2824	2720/2824	<10	21/2824	27/2824	29/2824	19/2824
		90.4 (89.4-91.5)		2.0 (1.5-2.5)	4.0 (3.2-4.7)	2.2 (1.7-2.8)	0.9 (0.5-1.2)	96.3 (95.6-97.0)		0.7 (0.4-1.1)	1.0 (0.6-1.3)	1.0 (0.7-1.4)	0.7 (0.4-1.0)
	White British	1970/2626	>=10	157/2626	320/2626	129/2626	37/2626	2289/2626	<10	59/2626	127/2626	98/2626	46/2626
		75.0 (73.4-76.7)		6.0 (5.1-6.9)	12.2 (10.9-13.4)	4.9 (4.1-5.7)	1.4 (1.0-1.9)	87.2 (85.9-88.4)		2.2 (1.7-2.8)	4.8 (4.0-5.7)	3.7 (3.0-4.5)	1.8 (1.3-2.3)
	Other	1570/1807	<10	63/1807	95/1807	56/1807	14/1807	1696/1807	<10	16/1807	34/1807	32/1807	24/1807
		86.9 (85.3-88.4)		3.5 (2.6-4.3)	5.3 (4.2-6.3)	3.1 (2.3-3.9)	0.8 (0.4-1.2)	93.9 (92.7-95.0)		0.9 (0.5-1.3)	1.9 (1.3-2.5)	1.8 (1.2-2.4)	1.3 (0.8-1.9)

		Playing cards						Bingo (elsewhere)					
		Never	Yes – NS	>1 year ago	This year	This month	This week	Never	Yes – NS	>1 year ago	This year	This month	This week
** Total**		6794/7257	18/7257	113/7257	193/7257	98/7257	41/7257	6882/7257	282/7257	28/7257	40/7257	17/7257	<10
		93.6 (93.1-94.2)	0.2 (0.1-0.4)	1.6 (1.3-1.8)	2.7 (2.3-3.0)	1.4 (1.1-1.6)	0.6 (0.4-0.7)	94.8 (94.3-95.3)	3.9 (3.4-4.3)	0.4 (0.2-0.5)	0.6 (0.4-0.7)	0.2 (0.1-0.3)	
Year Group	8	2353/2496	<10	29/2496	55/2496	39/2496	12/2496	2362/2496	115/2496	<10	<10	<10	<10
		94.3 (93.4-95.2)		1.2 (0.7-1.6)	2.2 (1.6-2.8)	1.6 (1.1-2.1)	0.5 (0.2-0.8)	94.6 (93.8-95.5)	4.6 (3.8-5.4)				
	9	2415/2583	<10	45/2583	70/2583	39/2583	13/2583	2458/2583	85/2583	13/2583	14/2583	<10	<10
		93.5 (92.5-94.4)		1.7 (1.2-2.2)	2.7 (2.1-3.3)	1.5 (1.0-2.0)	0.5 (0.2-0.8)	95.2 (94.3-96.0)	3.3 (2.6-4.0)	0.5 (0.2-0.8)	0.5 (0.3-0.8)		
	10	2026/2178	<10	39/2178	68/2178	20/2178	16/2178	2062/2178	82/2178	11/2178	18/2178	<10	<10
		93.0 (92.0-94.1)		1.8 (1.2-2.4)	3.1 (2.4-3.9)	0.9 (0.5-1.3)	0.7 (0.4-1.1)	94.7 (93.7-95.6)	3.8 (3.0-4.6)	0.5 (0.2-0.8)	0.8 (0.4-1.2)		
Sex	Female	3690/3889	<10	45/3889	94/3889	42/3889	11/3889	3666/3889	171/3889	12/3889	29/3889	<10	<10
		94.9 (94.2-95.6)		1.2 (0.8-1.5)	2.4 (1.9-2.9)	1.1 (0.8-1.4)	0.3 (0.1-0.5)	94.3 (93.5-95.0)	4.4 (3.8-5.0)	0.3 (0.1-0.5)	0.7 (0.5-1.0)		
	Male	3104/3368	>= 10	68/3368	99/3368	56/3368	30/3368	3216/3368	111/3368	16/3368	11/3368	<10	<10
		92.2 (91.3-93.1)		2.0 (1.5-2.5)	2.9 (2.4-3.5)	1.7 (1.2-2.1)	0.9 (0.6-1.2)	95.5 (94.8-96.2)	3.3 (2.7-3.9)	0.5 (0.2-0.7)	0.3 (0.1-0.5)		
Ethnicity	Pakistani British	2774/2824	<10	<10	16/2824	15/2824	<10	2803/2824	15/2824	<10	<10	<10	<10
		98.2 (97.7-98.7)			0.6 (0.3-0.8)	0.5 (0.3-0.8)		99.3 (98.9-99.6)	0.5 (0.3-0.8)				
	White British	2288/2626	>= 10	81/2626	155/2626	66/2626	24/2626	2312/2626	241/2626	27/2626	33/2626	11/2626	<10
		87.1 (85.8-88.4)		3.1 (2.4-3.7)	5.9 (5.0-6.8)	2.5 (1.9-3.1)	0.9 (0.6-1.3)	88.0 (86.8-89.3)	9.2 (8.1-10.3)	1.0 (0.6-1.4)	1.3 (0.8-1.7)	0.4 (0.2-0.7)	
	Other	1732/1807	<10	>= 10	22/1807	17/1807	>= 10	1767/1807	26/1807	<10	<10	<10	<10
		95.8 (94.9-96.8)			1.2 (0.7-1.7)	0.9 (0.5-1.4)		97.8 (97.1-98.5)	1.4 (0.9-2.0)				

		National Lottery games						Placing a bet					
		Never	Yes – NS	>1 year ago	This year	This month	This week	Never	Yes – NS	>1 year ago	This year	This month	This week
** Total**		6885/7257	14/7257	87/7257	152/7257	77/7257	42/7257	7106/7256	101/7256	11/7256	13/7256	13/7256	12/7256
		94.9 (94.4-95.4)	0.2 (0.1-0.3)	1.2 (0.9-1.4)	2.1 (1.8-2.4)	1.1 (0.8-1.3)	0.6 (0.4-0.7)	97.9 (97.6-98.3)	1.4 (1.1-1.7)	0.2 (0.1-0.2)	0.2 (0.1-0.3)	0.2 (0.1-0.3)	0.2 (0.1-0.3)
Year Group	8	2376/2496	<10	29/2496	45/2496	28/2496	12/2496	2439/2496	44/2496	<10	<10	<10	<10
		95.2 (94.4-96.0)		1.2 (0.7-1.6)	1.8 (1.3-2.3)	1.1 (0.7-1.5)	0.5 (0.2-0.8)	97.7 (97.1-98.3)	1.8 (1.2-2.3)				
	9	2437/2583	<10	33/2583	67/2583	25/2583	15/2583	2527/2583	36/2583	<10	<10	<10	<10
		94.3 (93.5-95.2)		1.3 (0.8-1.7)	2.6 (2.0-3.2)	1.0 (0.6-1.3)	0.6 (0.3-0.9)	97.8 (97.3-98.4)	1.4 (0.9-1.8)				
	10	2072/2178	<10	25/2178	40/2178	24/2178	15/2178	2140/2177	21/2177	<10	<10	<10	<10
		95.1 (94.2-96.0)		1.1 (0.7-1.6)	1.8 (1.3-2.4)	1.1 (0.7-1.5)	0.7 (0.3-1.0)	98.3 (97.8-98.8)	1.0 (0.6-1.4)				
Sex	Female	3717/3889	<10	45/3889	80/3889	32/3889	10/3889	3834/3888	38/3888	<10	<10	<10	<10
		95.6 (94.9-96.2)		1.2 (0.8-1.5)	2.1 (1.6-2.5)	0.8 (0.5-1.1)	0.3 (0.1-0.4)	98.6 (98.2-99.0)	1.0 (0.7-1.3)				
	Male	3168/3368	<10	42/3368	72/3368	45/3368	32/3368	3272/3368	63/3368	<10	<10	<10	<10
		94.1 (93.3-94.9)		1.2 (0.9-1.6)	2.1 (1.6-2.6)	1.3 (0.9-1.7)	1.0 (0.6-1.3)	97.1 (96.6-97.7)	1.9 (1.4-2.3)				
Ethnicity	Pakistani British	2770/2824	<10	14/2824	16/2824	11/2824	10/2824	2810/2823	<10	<10	<10	<10	<10
		98.1 (97.6-98.6)		0.5 (0.2-0.8)	0.6 (0.3-0.8)	0.4 (0.2-0.6)	0.4 (0.1-0.6)	99.5 (99.3-99.8)					
	White British	2407/2626	<10	49/2626	105/2626	35/2626	21/2626	2510/2626	85/2626	<10	>= 10	<10	<10
		91.7 (90.6-92.7)		1.9 (1.3-2.4)	4.0 (3.2-4.7)	1.3 (0.9-1.8)	0.8 (0.5-1.1)	95.6 (94.8-96.4)	3.2 (2.6-3.9)				
	Other	1708/1807	<10	24/1807	31/1807	31/1807	11/1807	1786/1807	<10	<10	<10	<10	<10
		94.5 (93.5-95.6)		1.3 (0.8-1.9)	1.7 (1.1-2.3)	1.7 (1.1-2.3)	0.6 (0.2-1.0)	98.8 (98.3-99.3)					

		Bingo (club)						Gaming machines					
		Never	Yes – NS	>1 year ago	This year	This month	This week	Never	Yes – NS	>1 year ago	This year	This month	This week
** Total**		7107/7257	10/7257	61/7257	42/7257	27/7257	10/7257	7145/7256	85/7256	<10	<10	<10	<10
		97.9 (97.6-98.3)	0.1 (0.0-0.2)	0.8 (0.6-1.1)	0.6 (0.4-0.7)	0.4 (0.2-0.5)	0.1 (0.0-0.2)	98.5 (98.2-98.8)	1.2 (0.9-1.4)				
Year Group	8	2446/2496	<10	19/2496	13/2496	13/2496	<10	2453/2496	35/2496	<10	<10	<10	<10
		98.0 (97.4-98.5)		0.8 (0.4-1.1)	0.5 (0.2-0.8)	0.5 (0.2-0.8)		98.3 (97.8-98.8)	1.4 (0.9-1.9)				
	9	2534/2583	<10	16/2583	17/2583	<10	<10	2543/2583	30/2583	<10	<10	<10	<10
		98.1 (97.6-98.6)		0.6 (0.3-0.9)	0.7 (0.3-1.0)			98.5 (98.0-98.9)	1.2 (0.8-1.6)				
	10	2127/2178	<10	26/2178	12/2178	<10	<10	2149/2177	20/2177	<10	<10	<10	<10
		97.7 (97.0-98.3)		1.2 (0.7-1.7)	0.6 (0.2-0.9)			98.7 (98.2-99.2)	0.9 (0.5-1.3)				
Sex	Female	3822/3889	<10	25/3889	21/3889	15/3889	<10	3846/3888	34/3888	<10	<10	<10	<10
		98.3 (97.9-98.7)		0.6 (0.4-0.9)	0.5 (0.3-0.8)	0.4 (0.2-0.6)		98.9 (98.6-99.2)	0.9 (0.6-1.2)				
	Male	3285/3368	<10	36/3368	21/3368	12/3368	<10	3299/3368	51/3368	<10	<10	<10	<10
		97.5 (97.0-98.1)		1.1 (0.7-1.4)	0.6 (0.4-0.9)	0.4 (0.2-0.6)		98.0 (97.5-98.4)	1.5 (1.1-1.9)				
Ethnicity	Pakistani British	2808/2824	<10	<10	<10	<10	<10	2779/2823	33/2823	<10	<10	<10	<10
		99.4 (99.2-99.7)						98.4 (98.0-98.9)	1.2 (0.8-1.6)				
	White British	2513/2626	<10	44/2626	36/2626	21/2626	<10	2590/2626	30/2626	<10	<10	<10	<10
		95.7 (94.9-96.5)		1.7 (1.2-2.2)	1.4 (0.9-1.8)	0.8 (0.5-1.1)		98.6 (98.2-99.1)	1.1 (0.7-1.6)				
	Other	1786/1807	<10	>= 10	<10	<10	<10	1776/1807	22/1807	<10	<10	<10	<10
		98.8 (98.3-99.3)						98.3 (97.7-98.9)	1.2 (0.7-1.7)				

		Gaming websites/apps						Casino					
		Never	Yes – NS	>1 year ago	This year	This month	This week	Never	Yes – NS	>1 year ago	This year	This month	This week
** Total**		7165/7255	49/7255	<10	11/7255	<10	15/7255	7227/7254	15/7254	<10	<10	<10	<10
		98.8 (98.5-99.0)	0.7 (0.5-0.9)		0.2 (0.1-0.2)		0.2 (0.1-0.3)	99.6 (99.5-99.8)	0.2 (0.1-0.3)				
Year Group	8	2475/2496	14/2496	<10	<10	<10	<10	2489/2495	<10	<10	<10	<10	<10
		99.2 (98.8-99.5)	0.6 (0.3-0.9)					99.8 (99.6-99.9)					
	9	2549/2582	15/2582	<10	<10	<10	<10	2571/2582	<10	<10	<10	<10	<10
		98.7 (98.3-99.2)	0.6 (0.3-0.9)					99.6 (99.3-99.8)					
	10	2141/2177	20/2177	<10	<10	<10	<10	2167/2177	<10	<10	<10	<10	<10
		98.3 (97.8-98.9)	0.9 (0.5-1.3)					99.5 (99.3-99.8)					
Sex	Female	3865/3888	10/3888	<10	<10	<10	<10	3878/3888	<10	<10	<10	<10	<10
		99.4 (99.2-99.6)	0.3 (0.1-0.4)					99.7 (99.6-99.9)					
	Male	3300/3367	39/3367	<10	<10	<10	>= 10	3349/3366	<10	<10	<10	<10	<10
		98.0 (97.5-98.5)	1.2 (0.8-1.5)					99.5 (99.3-99.7)					
Ethnicity	Pakistani British	2805/2822	11/2822	<10	<10	<10	<10	2808/2821	<10	<10	<10	<10	<10
		99.4 (99.1-99.7)	0.4 (0.2-0.6)					99.5 (99.3-99.8)					
	White British	2575/2626	27/2626	<10	<10	<10	<10	2621/2626	<10	<10	<10	<10	<10
		98.1 (97.5-98.6)	1.0 (0.6-1.4)					99.8 (99.6-100.0)					
	Other	1785/1807	11/1807	<10	<10	<10	<10	1798/1807	<10	<10	<10	<10	<10
		98.8 (98.3-99.3)	0.6 (0.2-1.0)					99.5 (99.2-99.8)					

Distinct patterns by sex and ethnicity were observed. Overall participation in gambling was similar between males (29.6%) and females (28.0%). However, males were more likely than females to have participated in several specific forms of gambling, including National Lottery games (6.7% vs 4.6%, Figure 1); private bets (10.5% vs 5.6%, Figure 2); playing cards for money (8.2% vs 5.4%, Figure 3); placing bets in betting shops (3.1% vs 1.6%, Figure 4); using gaming machines in betting shops (2.4% vs 1.2%, Figure 5); and using gambling websites/apps (2.3% vs 0.7%, Figure 6).

In contrast, playing on fruit or slot machines was more likely in females (19.0% vs 14.0%, Figure 7). Playing bingo was similar between sexes and very few participants had spent money in casinos ( Figures 8- 10).

The most pronounced differences were observed across the pre-defined ethnic groups. Pupils of White British ethnicity reported markedly higher rates of ever spending money on gambling (45.8%) compared to pupils of Pakistani British ethnicity (15.8%) and other ethnic groups (24.1%). This disparity was evident across most activities. For example, playing on fruit or slot machines was reported by 25.6% of White British pupils compared to 10.2% of pupils of Pakistani British ethnicity ( Figure 7). Similarly large differences were seen for lotto (8.7% vs 2.5%, Figure 1), private bets (13.2% vs. 3.9%, Figure 2), playing cards for money (13.3% vs. 1.9%, Figure 3), placing bets in betting shops (4.6% vs 0.7%, Figure 4), playing bingo at a bingo club (4.4% vs 0.7%, Figure 8) or elsewhere (12.1% vs 0.9%, Figure 9), and using gambling websites/apps (2.1% vs 0.9%, Figure 6).

### In-game spending and gambling-like activities

The survey also examined behaviours that combine gaming and potential gambling-like activity. Figure 11 shows the prevalence of spending money within online games, stratified by demographic group. Table 6 provides detailed counts and prevalence estimates (with 95% confidence intervals) for in-game spending and gambling-like activities.

**Table 6.** Prevalence of spending money in online games and engagement with gambling-like features, by school year group, sex, and ethnicity.

Group	Level	At least one	Purchased in-game items	Loot boxes and packs	Bet with in-game items
**Total**		**4364/7828**	**4116/7828**	**2126/7828**	**255/7828**
		**55.8 (54.6-56.9)**	**52.6 (51.5-53.7)**	**27.2 (26.2-28.1)**	**3.3 (2.9-3.6)**
Year Group	8	1517/2684	1419/2684	701/2684	72/2684
		56.5 (54.6-58.4)	52.9 (51.0-54.8)	26.1 (24.5-27.8)	2.7 (2.1-3.3)
	9	1600/2807	1512/2807	795/2807	94/2807
		57.0 (55.2-58.8)	53.9 (52.0-55.7)	28.3 (26.6-30.0)	3.4 (2.7-4.0)
	10	1247/2337	1185/2337	630/2337	89/2337
		53.4 (51.3-55.4)	50.7 (48.7-52.7)	27.0 (25.2-28.8)	3.8 (3.0-4.6)
Sex	Female	1629/4093	1536/4093	652/4093	67/4093
		39.8 (38.3-41.3)	37.5 (36.1-39.0)	15.9 (14.8-17.0)	1.6 (1.2-2.0)
	Male	2735/3735	2580/3735	1474/3735	188/3735
		73.2 (71.8-74.7)	69.1 (67.6-70.6)	39.5 (37.9-41.0)	5.0 (4.3-5.7)
Ethnicity	Pakistani British	1438/3103	1357/3103	620/3103	67/3103
		46.3 (44.6-48.1)	43.7 (42.0-45.5)	20.0 (18.6-21.4)	2.2 (1.7-2.7)
	White British	1851/2773	1756/2773	980/2773	111/2773
		66.8 (65.0-68.5)	63.3 (61.5-65.1)	35.3 (33.6-37.1)	4.0 (3.3-4.7)
	Other	1075/1952	1003/1952	526/1952	77/1952
		55.1 (52.9-57.3)	51.4 (49.2-53.6)	27.0 (25.0-28.9)	3.9 (3.1-4.8)

Overall, representing the population-level prevalence across all surveyed pupils, a majority of pupils (55.8%) reported having spent real money in at least one online game. A substantial minority (over 25%) had paid to open “loot boxes” or similar randomised reward packs, a mechanism with similarities to gambling. A smaller group (less than 5%) reported using in-game items to bet on external websites, a form of unregulated gambling.

These in-game spending behaviours were broadly similar between year groups 8, 9, and 10.

The most pronounced differences were observed by sex and ethnicity. Males were substantially more likely than females to have spent money on at least one online game (73.2% vs 39.8%). Males were also more likely to have spent money to buy in-game items (69.1% vs 37.5%) and to purchase loot boxes (39.5% vs 15.9%). A marked disparity was also seen between ethnic groups, with pupils of White British ethnicity reporting the highest rates of spending money on at least one online game (66.8%), followed by pupils of other ethnicities (55.1%), and then pupils of Pakistani British ethnicity (46.3%).

## Discussion

In this survey of over 7,000 Bradford teenagers, we found that more than one in four had spent their own money on gambling activities. This figure is similar to the UK national average of 27% reported by the 2024 Gambling Commission, suggesting that Bradford’s young people engage in similar levels of gambling activity.

The most pronounced finding was the disparity in gambling prevalence by ethnicity, with pupils of White British ethnicity being almost three times more likely to have gambled than their peers of Pakistani British ethnicity across a range of activities. The reasons for these findings are likely complex and may involve a combination of cultural, social, and religious factors that may influence both participation and reporting but that we were unable to directly measure. This substantial difference suggests that broad, one-size-fits-all public health campaigns may be insufficient.

Our data for in-game spending, where over half of pupils have paid for items and over a quarter have opened loot boxes, suggest some support for the growing concern about the blurring lines between gaming and gambling. These “gambling-like” mechanisms may act as a gateway by normalising the mechanics of chance and reward for young people, warranting further investigation and potential policy action. ^10^

### Strengths and limitations

The primary strength of this study is its large, diverse, community-based sample, providing a unique insight into a young, multi-ethnic urban population. However, the study has limitations. First, the data is self-reported and may be subject to recall or social desirability bias. Second, the online gaming survey question did not include a preceding screening question to establish baseline gaming participation. Consequently, respondents who selected ‘None of these’ regarding types of in-game purchase could be both true non-gamers but could also be active gamers for whom there was either not a suitable option for their type of in-game spend or who engaged exclusively with ‘free-to-play’ mechanics. Because these groups cannot be disaggregated, our estimates represent the prevalence of in-game spending among all survey participants rather than the specific prevalence among those who reported being active gamers. Therefore, the estimates of spending prevalence among those who are active gamers may be higher than that reported here for all participants. Third, the cross-sectional design means we can identify associations but cannot infer causality or track the progression of gambling behaviours over time. Finally, the information is based on responses from a subset of schools and pupils in Bradford and is not a random sample, so findings may not be generalisable to other populations.

## Conclusion

These data suggest that gambling is a common activity among Bradford’s young people. Our data are available on request to support the development of a stronger evidence base around young people’s gambling that can help inform the development of targeted interventions and school-based education programmes that are culturally sensitive and address the specific gambling behaviours prevalent in this age group, as well as the potential convergence of gaming and gambling.

## Data Availability

Individual-level data from Born in Bradford study cannot be made publicly available due to ethical approvals and the need to protect participant confidentiality. Researchers may apply to access these data via the Born in Bradford Data Access process. Full details of the application process, eligibility criteria, and data access conditions are available at https://borninbradford.nhs.uk/our-data/how-to-access-data/. Applications are reviewed subject to appropriate scientific justification, governance approval, and completion of a data sharing agreement. Enquiries can be directed to the Born in Bradford research team ( borninbradford@bthft.nhs.uk). Age of Wonder 2023-24 questionnaires, information on differences between survey responders and eligible non-responders, and R code for producing tables and figures are available via the Open Science Framework (OSF): **10.17605/OSF.IO/A2ZB4** ^11^ These data are available under the Creative Commons CC0 1.0 Universal licence.
