# Supplementary material for: Gambling behaviour of Bradford teenagers: evidence from the Born in Bradford Age of Wonder study
Source: Wellcome Open Res. 2026 Aug 17;11:112. Originally published 2026 Feb 14. [Version 2] doi: 10.12688/wellcomeopenres.25913.2 (PMC13654074; doi:10.12688/wellcomeopenres.25913.2)
Supplement: Peer review history

# Reviewer report: Reviewer response for version 2

**DOI:** 10.21956/wellcomeopenres.29428.r164720

**Published:** 2026-10-12

**Authors:** Beth T. Bell

**Affiliations:** 1 University of York, York, England, UK

This looks great. I think I may have left this comment as a response as I hadn't logged into the system properly, but the abstract and table 3 still report two different figures for respondents to gambling questions: 7,257/7,828 (methods section of abstract) and  7,254/7,825 (results). I can't where this discrepancy is explained.

Are sufficient details of methods and materials provided to allow replication by others?

Yes

Is the rationale for creating the dataset(s) clearly described?

Yes

Are the datasets clearly presented in a useable and accessible format?

Partly

Are the protocols appropriate and is the work technically sound?

Yes

Reviewer Expertise:

Adolescents' digital technology use and wellbeing

I confirm that I have read this submission and believe that I have an appropriate level of expertise to confirm that it is of an acceptable scientific standard.

# Reviewer report: Reviewer response for version 2

**DOI:** 10.21956/wellcomeopenres.29428.r169191

**Published:** 2026-10-10

**Authors:** Elvira Bolat

**Affiliations:** 1 Bournemouth University, Bournemouth, UK

The study addresses an important public health issue and offers a valuable contribution through its large, ethnically diverse adolescent sample and its inclusion of both conventional gambling and gaming-related expenditure.

 My assessment focuses on the scientific robustness of the dataset, transparency of the data collection and cleaning procedures, consistency of the reported findings, and accessibility and reproducibility of the underlying data.

 While the dataset has considerable potential for informing future research, I identify several methodological and reporting issues that require clarification, particularly concerning analytical denominators, participant exclusions, conceptual distinctions between gaming expenditure and gambling-related behaviours, and documentation supporting reproducibility. 

**Essential points to address (1-5):**

 1. Correct inconsistent sample sizes and denominators. The manuscript reports 7,257 and 7,828 participants in some sections, but 7,254 and 7,825 elsewhere. The authors must reconcile these figures across the text, tables and figures, and explain any differences arising from missing data.

 2. Justify the exclusion of respondents selecting all gambling activities. The authors assume these responses are invalid, but some adolescents may genuinely participate in multiple activities. They should report how many participants were excluded under each criterion and ideally conduct a sensitivity analysis to establish whether exclusions affect prevalence estimates.

 3. Distinguish gambling-like mechanisms from ordinary gaming expenditure. Purchasing specific in-game items, buying randomised loot boxes and betting with virtual items are conceptually different behaviours. The authors should avoid treating all in-game spending as gambling-like activity and clarify exactly what each prevalence estimate represents.

 4. Improve methodological reproducibility. The authors should document the coding of multiple-response questions, missing-data treatment, construction of composite indicators and calculation of prevalence estimates. They should also confirm that the available R code and data dictionary allow approved researchers to reproduce the reported analyses.

 5. Clarify the population used to calculate gaming related prevalence. The survey does not establish which respondents actively play games. Therefore, the reported prevalence reflects expenditure among survey respondents, not among active gamers. This distinction must remain consistent throughout the manuscript.

**Additional recommendations (6-8):**

 6. Interpret ethnic differences cautiously. The substantial differences between White British and Pakistani British respondents warrant attention, but ethnicity alone cannot explain these patterns. The authors should recognise potential socioeconomic, cultural, religious and reporting-related influences.

 7. Avoid implying a causal gateway from gaming to gambling. The cross-sectional data cannot demonstrate that loot box engagement leads to subsequent gambling. Such relationships should be presented as questions for future longitudinal research.

 8. Strengthen the discussion of representativeness. Only 25 of 36 invited schools participated, and the reported overall response rate was 40.9%. The authors should discuss possible non-response and selection biases and qualify the generalisability of their estimates.

Are sufficient details of methods and materials provided to allow replication by others?

Partly

Is the rationale for creating the dataset(s) clearly described?

Yes

Are the datasets clearly presented in a useable and accessible format?

Partly

Are the protocols appropriate and is the work technically sound?

Partly

Reviewer Expertise:

Digital marketing and consumer behaviour; gambling marketing and gambling-related harms among children and young people; gaming-gambling convergence; digital platforms, gamification and dark patterns; commercial determinants of health; and responsible digital marketing.

I confirm that I have read this submission and believe that I have an appropriate level of expertise to confirm that it is of an acceptable scientific standard, however I have significant reservations, as outlined above.

# Reviewer report: Reviewer response for version 2

**DOI:** 10.21956/wellcomeopenres.29428.r164721

**Published:** 2026-09-10

**Authors:** Jess Staphanous

**Affiliations:** 1 Anna Freud, London, UK

Many thanks for the revisions. I have no further comments.

Are sufficient details of methods and materials provided to allow replication by others?

Yes

Is the rationale for creating the dataset(s) clearly described?

Yes

Are the datasets clearly presented in a useable and accessible format?

Yes

Are the protocols appropriate and is the work technically sound?

Yes

Reviewer Expertise:

Child and adolescent mental health

I confirm that I have read this submission and believe that I have an appropriate level of expertise to confirm that it is of an acceptable scientific standard.

# Reviewer report: Reviewer response for version 1

**DOI:** 10.21956/wellcomeopenres.28523.r153312

**Published:** 2026-04-28

**Authors:** Beth T. Bell

**Affiliations:** 1 University of York, York, England, UK

The article presents cross-sectional survey data from the Born in Bradford Age of Wonder study, collected during the 2023–24 academic year. It  presents prevalence rates for gambling behaviour and gambling-like gaming behaviours (e.g., loot box use) among adolescents in school years 8–10. Results are stratified by year group, sex, and ethnicity, with a focus on White British, Pakistani British, and “Other” ethnic categories.

 I have some small suggestions to further enhance clarity:

 - Add more detail on the decision to exclude those who selected "all options in the affirmative" - I'm not sure I fully understand this or the underlying rationale.

 - The denominators in  analyses need to be clarified. Unless I have misunderstood, the article states that the analytic sample for traditional gambling was n = 7,254, yet tables use different totals (e.g., fruit/slot machines denominator = 7,257).

 - Please add a reference to support the claim that there are mounting concerns about gambling-like features in games acting as a "gateway" to gambling.

Are sufficient details of methods and materials provided to allow replication by others?

Yes

Is the rationale for creating the dataset(s) clearly described?

Yes

Are the datasets clearly presented in a useable and accessible format?

Partly

Are the protocols appropriate and is the work technically sound?

Yes

Reviewer Expertise:

Adolescents' digital technology use and wellbeing

I confirm that I have read this submission and believe that I have an appropriate level of expertise to confirm that it is of an acceptable scientific standard, however I have significant reservations, as outlined above.

# Response

**Published:** 2026-05-06

**Authors:** Gillian Santorelli

**Affiliations:** Bradford Hospitals National Health Service Trust, UK

**Reviewer 2:**

1. **Add more detail on the decision to exclude those who selected "all options in the affirmative" - I'm not sure I fully understand this or the underlying rationale. **

**Response: **Thank you for this feedback, which was also echoes by Reviewer 1. We have now expanded the Methods section to explicitly justify this decision: “These responses were considered implausible and indicative of non-differentiation (i.e., selecting all items without regard to content), a recognised indicator of low-quality survey data.” (ref: Cornell et al., 2012) to support excluding these implausible responses to ensure data validity.

1. **The denominators in analyses need to be clarified. Unless I have misunderstood, the article states that the analytic sample for traditional gambling was n = 7,254, yet tables use different totals (e.g., fruit/slot machines denominator = 7,257). **

**Response: **Thank you for highlighting this discrepancy. This was a typographic error. We have reviewed the data and aligned all denominators in the tables to accurately reflect the true analytic sample.

1. **Please add a reference to support the claim that there are mounting concerns about gambling-like features in games acting as a "gateway" to gambling.**

**Response: **We have added the following reference: Zendle D. Beyond loot boxes: a variety of gambling-like practices in video games are linked to both problem gambling and disordered gaming. PeerJ. 2020 Jul 14;8:e9466. doi: 10.7717/peerj.9466. PMID: 32742782; PMCID: PMC7367055. In addition, the Gambling commission has selected this as a theme in their Statistics and Research programme.

# Reviewer report: Reviewer response for version 1

**DOI:** 10.21956/wellcomeopenres.28523.r151637

**Published:** 2026-03-31

**Authors:** Jess Staphanous

**Affiliations:** 1 Anna Freud, London, UK

Many thanks for the opportunity to review this paper on a topic that will only grow in its importance. This paper describes gambling behaviours from pupils in Years 8-10 in the Born in Bradford Age of Wonder study. Responses to a variety of questions were summarised by year group, sex, and ethnicity.

 Please see specific comments below:

 - The rationale for excluding participants who selected all options in the affirmative could be made explicit.

 - Figure 1 title is cut off.

 - Figure 6 title appears to be missing a word (apps where you can...?)

 - Figure 10 doesn't appear to be useful.

 It’s unclear what the denominator is for the prevalence of spending money in online games. The table appears to use all respondents who answered the online gaming questions (N = 7,825), but this may inflate or distort the estimate because not all respondents actually play online games. Given that gaming participation differs by gender, it would be helpful to report spending prevalence among gamers only. Also, there is a small discrepancy in the Ns between Table 2 and Table 5 (7,825 vs. 7,828), which may need clarification.

 - For the comparisons between groups (e.g. ethnicity) were statistical tests conducted to determine whether these differences were statistically significant?

 - I note that the data were collected during a period of increased policy attention to gambling‑like features in online games. The UK Government published its response to the loot box call for evidence in July 2022, and industry‑led principles coordinated by Ukie were introduced in July 2023 with Government support. These developments form part of the broader context in which young people’s online gaming and spending behaviours should be interpreted.

Are sufficient details of methods and materials provided to allow replication by others?

Yes

Is the rationale for creating the dataset(s) clearly described?

Yes

Are the datasets clearly presented in a useable and accessible format?

Yes

Are the protocols appropriate and is the work technically sound?

Yes

Reviewer Expertise:

Child and adolescent mental health

I confirm that I have read this submission and believe that I have an appropriate level of expertise to confirm that it is of an acceptable scientific standard, however I have significant reservations, as outlined above.

# Response

**Published:** 2026-05-06

**Authors:** Gillian Santorelli

**Affiliations:** Bradford Hospitals National Health Service Trust, UK

We are grateful to the reviewers for their time and insightful feedback, which has helped us improve the clarity and quality of our manuscript. Please find our detailed, point-by-point responses to each comment below. 

**Comment:** **The rationale for excluding participants who selected all options in the affirmative could be made explicit.**

**Response: **We thank the reviewer for highlighting this and agree it required clarification. We have now expanded the Methods section to explicitly justify this decision: “These responses were considered implausible and indicative of non-differentiation (i.e., selecting all items without regard to content), a recognised indicator of low-quality survey data.” (ref: Cornell et al., 2012) to support excluding these implausible responses to ensure data validity.

**Comment:** **Figure 1 title is cut off.**

**Response: **Thank you for bringing this to our attention. We have now corrected this.

**Comment:** **Figure 6 title appears to be missing a word (apps where you can...?)**

**Response: **Again, thank you for bringing this to our attention. We have now corrected this.

**Comment:** **Figure 10 doesn't appear to be useful.**

**Response: **We appreciate the reviewer's observation. We agree that the near-zero prevalence of casino gambling makes the figure visually uninteresting from an analytical standpoint. However, because this manuscript is submitted as a Data Note, our primary objective is to comprehensively catalogue the dataset and share early findings rather than solely report significant findings. We have retained Figure 10 to maintain consistency across all the traditional gambling questions included in the survey. This ensures that future researchers have a complete and transparent overview of the variables available for secondary analysis, even those with low prevalence.

**​​​​​​​Comment:** **It’s unclear what the denominator is for the prevalence of spending money in online games. The table appears to use all respondents who answered the online gaming questions (N = 7,825), but this may inflate or distort the estimate because not all respondents actually play online games. Given that gaming participation differs by gender, it would be helpful to report spending prevalence among gamers only. Also, there is a small discrepancy in the Ns between Table 2 and Table 5 (7,825 vs. 7,828), which may need clarification.**

**Response: **Thank you for highlighting this important point. First, we have corrected the typographical error regarding the sample size; the correct denominator is 7,825, and this has now been aligned consistently throughout the text and tables. Regarding the denominator for online gaming prevalence, we entirely agree that reporting spending prevalence exclusively among active gamers would be highly valuable. However, the survey module did not include a preceding screening question to establish baseline gaming participation. The question asked respondents to select all that apply from four options:

1. Paid money to buy specific in-game items
2. Paid money to open loot boxes/packs/chests
3. Bet with in-game items
4. None of these

Consequently, the “None of these” group inherently conflates two distinct groups: true non-gamers, and active gamers for whom none of the first three options were applicable as well as gamers who play 'free-to-play' games without spending money. Because we cannot separate these groups within the data, we are unable to calculate a true 'gamers-only' denominator (as excluding the "None of these" group entirely would artificially inflate the spending prevalence to 100%). We acknowledge this limitation and have revised the text to clarify that our estimates represent the *prevalence* among all respondents providing an estimate of in-game spending among Bradford teenagers rather than the prevalence specifically among gamers. Furthermore, we have explicitly added the lack of a gaming screening question, and the resulting ambiguity of the 'None of these' response, to the Limitations section of the Discussion. Additionally, to provide full transparency regarding the survey design, we have added a new table detailing the exact wording of all gambling and gaming questions and their corresponding response options.

**​​​​​​​Comment:** **For the comparisons between groups (e.g. ethnicity) were statistical tests conducted to determine whether these differences were statistically significant?**  

**Response: **We appreciate the reviewer highlighting this. While establishing statistical significance between these demographic groups is an important next step, we have deliberately chosen not to conduct formal statistical tests in this manuscript. As a Data Note, the purpose of this paper is to provide a descriptive overview of the data collected rather than a full, hypothesis-driven analysis. We felt that running raw statistical tests without controlling for complex confounders could lead to misinterpretation. Instead, our goal is to present this baseline data to the academic community so that future researchers can make use of our data in protocol-driven robust academic studies.  

**​​​​​​​Comment:** **I note that the data were collected during a period of increased policy attention to gambling‑like features in online games. The UK Government published its response to the loot box call for evidence in July 2022, and industry‑led principles coordinated by Ukie were introduced in July 2023 with Government support. These developments form part of the broader context in which young people’s online gaming and spending behaviours should be interpreted.**

**Response: **​​​​​​​We strongly agree with the reviewer that the policy backdrop provides important context for interpretation of research findings. Again, what we present here is a data note showcasing the Age of Wonder data and our early findings relating to gambling in order to promote use of the data among the wider academic community. We present simple descriptive data with limited interpretation and management of confounders and potential alternative explanations for our findings in the way we would within a full protocol driven research study. As such, we have not explored the wider political context at the time these data were collected but would absolutely encourage that should this data be used within a comprehensive protocol driven study.
